# Selective Pressures Influence a Grow Fast, Fly Young Strategy for Black Skimmer Populations in the Peruvian Amazon

**DOI:** 10.1002/ece3.73676

**Published:** 2026-05-18

**Authors:** Katharine S. Goodenough, Torbjørn Haugaasen, Eli S. Bridge

**Affiliations:** ^1^ School of Biological Sciences, Dodge Family College of Arts and Science University of Oklahoma Norman Oklahoma USA; ^2^ Oklahoma Biological Survey Norman Oklahoma USA; ^3^ Faculty of Environmental Sciences and Natural Resource Management Norwegian University of Life Sciences Ås Norway

**Keywords:** flood threat, growth rate, life history variation, nest predation, reproductive success, waterbird

## Abstract

Black Skimmers ( *Rynchops niger* ) typically nest in coastal environments. However, there are populations in South America that nest along freshwater river tributaries, where they are subjected to threats from predation and flooding that differ from coastal counterparts. We monitored Black Skimmers from a population in Manu National Park, Peru, over two breeding seasons and compared their life history traits with those of Black Skimmer populations across the Americas. Mean clutch size, daily nest survival rate, hatching success, and incubation period were within reported ranges. However, incubation bouts were short (7.33 ± 1.84 h^−1^), provisioning rates were high (3.58 ± 1.35 prey items chick^−1^ h^−1^), and chicks fledged about 10 days earlier than typically reported in North American populations. Flooding accounted for 41%–61% of nest failures, concentrating mortality risk during early development. Across populations, initial reproductive investment appears conserved, whereas traits influencing the duration of offspring vulnerability varied. High food availability coupled with a tenuous nesting situation appears to select for individuals that exploit abundant food resources and maximize chick growth rates, such that a shortened growth period may limit exposure to stage‐specific mortality influences.

## Graphical abstract

This article provides reproductive data for a population of Black Skimmers nesting in the Peruvian Amazon and compares the results of the Manu data with published literature in Brazil and North America.

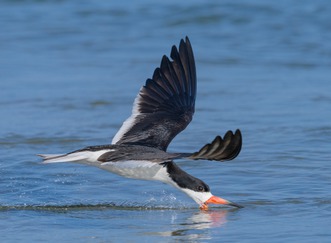

## 1 Introduction

Life‐history strategies describe how organisms resolve allocation tradeoffs among reproduction, maintenance, and survival under variable environmental conditions (Stearns and Koella 1986; Stearns 1989, 1992; Ricklefs and Wikelski 2002; Martin et al. 2006). Because mortality risk and resource availability often fluctuate within and across breeding seasons, selection is expected to favor flexible strategies that allow individuals to adjust reproductive effort in ways that mitigate stage‐specific threats (Bonsall and Klug 2011). Phenotypic plasticity in reproductive traits such as clutch size, incubation behavior, provisioning rates, and offspring growth trajectories can permit parents to alter investment patterns in response to predictable or stochastic environmental pressures (Lima and Dill 1990; Arendt 1997; Ghalambor and Martin 2002; Jetz et al. 2008; Wingfield 2013, Royle et al. 2014). When mortality risk is concentrated in a particular developmental window, plastic shifts that reduce the duration of vulnerability during this time frame may enhance fitness (Ghalambor and Martin 2002).

For birds, reproductive tradeoffs are often expressed through adjustments in parental behavior. Chick growth and survival are commonly associated with local prey availability and linked with variation in parental care behaviors such as offspring provisioning (Piatt et al. 2007; Royle et al. 2014; Vincze et al. 2017). Adults may modify the timing and duration of foraging, incubation bout length, nest attendance, or chick provisioning rates depending upon food availability, predation risk, and weather conditions (Martin 1987, 1995, 1996; Lima 2009; Martin et al. 2011; Royle et al. 2014; Vincze et al. 2017; Reger et al. 2018). The predictability and composition of selective threats influencing breeding birds can influence how these tradeoffs are resolved (Hunter et al. 2016). For example, when nest predation risk is high, parents may alter incubation activities or nest location selection to reduce detectability (Lima 2009; Martin et al. 2011). Alternatively, when mortality risk is driven by episodic events such as flooding, selection may favor strategies that accelerate offspring development, decreasing the time period during which nests or chicks remain vulnerable to catastrophic loss. However, sustaining the elevated provisioning rates needed to drive faster chick growth requires greater parental energetic expenditure, potentially at the cost of parental body condition or future reproductive success. Biparental care strategies, through coordinated efforts in parental behavior and offspring growth, represent a mechanism by which phenotypic plasticity could mediate these competing demands by balancing parental self‐maintenance against offspring investment, and minimizing the duration of stage‐specific vulnerability against the energetic costs of accelerated development (Lima 2009; Martin et al. 2011).

In many ecosystems, flooding constitutes a catastrophic but temporally constrained threat capable of destroying large numbers of nests within a single event (Van De Pol et al. 2010; Bailey et al. 2017). If flood risk is recurrent during the nesting period, the duration of the chick rearing stage may become a critical determinant of offspring survival. Under these environments, elevated chick provisioning rates and accelerated chick growth could reduce cumulative exposure to mortality risk if species are able to perceive cumulative risks. Conversely, in environments where food is limiting or predation risk is elevated, parents may be constrained in their ability to increase parental investment, which could result in slower growth or prolonged developmental periods (Metcalfe and Monaghan 2001).

**FIGURE 1:**
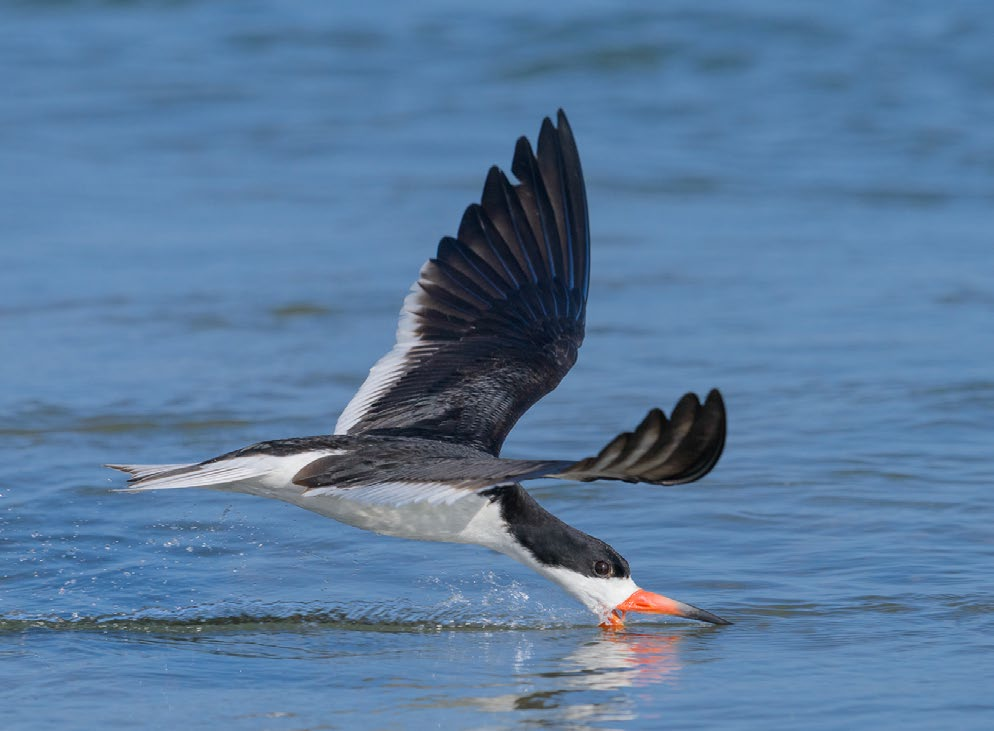
A photograph of a coastal Black Skimmer *Rynchops niger* foraging in San Diego Bay, San Diego, California taken by Bruno Enrique Struck (2016).

Colonial nesting waterbirds provide a compelling system in which to examine these dynamics because reproductive success is frequently influenced by pressures acting collectively, for example, nest predation, food limitation, extreme temperatures, and flooding. The Black Skimmer ( *Rynchops niger* ) is a colonial nesting waterbird (Figure 1) with a breeding range that spans the Americas from 40° N to 38° S (Vieira et al. 2018). Skimmers employ a characteristic style of foraging that is highly reliant upon fish near the water surface (Zusi 1962; Blake 1985), and they typically nest on open beaches or sandy river banks or river islands. Although skimmers are active throughout the day, their foraging activities are mainly crepuscular or nocturnal, with their tactile feeding allowing them to catch fish successfully in low light (Rojas et al. 1997; Gochfeld et al. 2020).

In North America, the Black Skimmer subspecies *R.n. niger* is predominantly a coastal species for the entirety of the annual cycle, whereas in South America many populations (*R.n. cinerascens* and *R.n. intercedens*) nest inland in freshwater river and lake systems before migrating to coastal areas (Davenport et al. 2016; Gochfeld et al. 2020). Food availability for skimmer populations varies from patchily distributed marine‐based resources (Erwin 1977a, 1977b; Erwin and Korschgen 1979; Gordon et al. 2000) to abundant slow river freshwater resources (Mariano‐Jelicich et al. 2014). Hence, food availability likely influences several aspects of life history for the Black Skimmer.

Although the suite of selective pressures experienced by Black Skimmer populations is broadly similar across their range—nest predation, flooding, thermal stress, and variation in food availability—the intensity and timing of these pressures differ among regions, although this has not been formally tested. Coastal colonies frequently experience nest loss associated with predation, storm tides, and perigean spring tides (Burger 1982; Maslo et al. 2016), while inland riverine populations may be subject to unpredictable flash flooding associated with localized storm systems. Additionally, cold‐air incursions from the Andes (friajes) can generate abrupt increases in river discharge during the dry season, inundating exposed riverbanks used for nesting (Marengo et al. 1997; Garreaud 1999). Both localized rain storms and friaje events may repeatedly reset breeding attempts within a season. At the same time, white‐water rivers draining Andean headwaters can support high aquatic productivity, potentially providing abundant food resources near nesting sites (Fittkau et al. 1975; Osorio et al. 2011). The combination of elevated flood risk and high prey availability creates an ecological context in which accelerated offspring development may be both feasible and advantageous.

Variation in chick growth rates among avian populations is often associated with differences in parental provisioning and local prey conditions (Martin 1995, 1996; Piatt et al. 2007; Lima 2009; Martin et al. 2011; Royle et al. 2014; Vincze et al. 2017). Growth trajectories in birds appear to be flexible rather than fixed, with evidence that offspring can grow at faster rates when selection favors rapid attainment of a size or developmental stage that confers increased survival (Ricklefs 1969; Schew and Ricklefs 1998; Metcalfe and Monaghan 2001). Because the Black Skimmer exhibits biparental care and observable provisioning behavior, it offers an opportunity to evaluate whether parental investment and chick growth vary in a manner consistent with risk‐sensitive reproductive provisioning.

Population‐level differences in parental behavior and offspring growth can arise through two non‐exclusive mechanisms: phenotypic plasticity, in which individuals can alter investment in response to local conditions via a shared reaction norm, or local adaptation, in which divergent selection favors fixed trait differences among populations with distinct evolutionary histories (Stearns and Koella 1986; Westneat et al. 2011). Distinguishing between these requires common‐garden or reciprocal transplant experiments that are logistically difficult for highly mobile species like skimmers. Nevertheless, comparing life‐history traits across populations can help reveal whether observed patterns are better explained by one mechanism or the other, and weighing that evidence is essential for understanding why individuals within the same species vary in their reproductive strategies.

In this study, we examine reproductive, behavioral, and developmental traits of a Black Skimmer population breeding along the Manu River in the Madre de Dios Province, Peru and compare these traits with published data from other Black Skimmer populations across the Americas. The Manu River population nests along exposed riverbanks during the dry season, where flash flooding constitutes a recurrent source of nest failure. We quantify clutch size, incubation behavior, provisioning rates, chick growth trajectories, and age at fledging for the Manu population, and we evaluate these traits in the context of regional variation in mortality sources. By comparing life‐history traits across ecological contexts, we assess whether patterns observed in the Manu population are consistent with phenotypic plasticity that enables Black Skimmers to resolve reproductive trade‐offs under seasonally shifting threats.

## 2 Methods

### 2.1 Study Area

**FIGURE 2:**
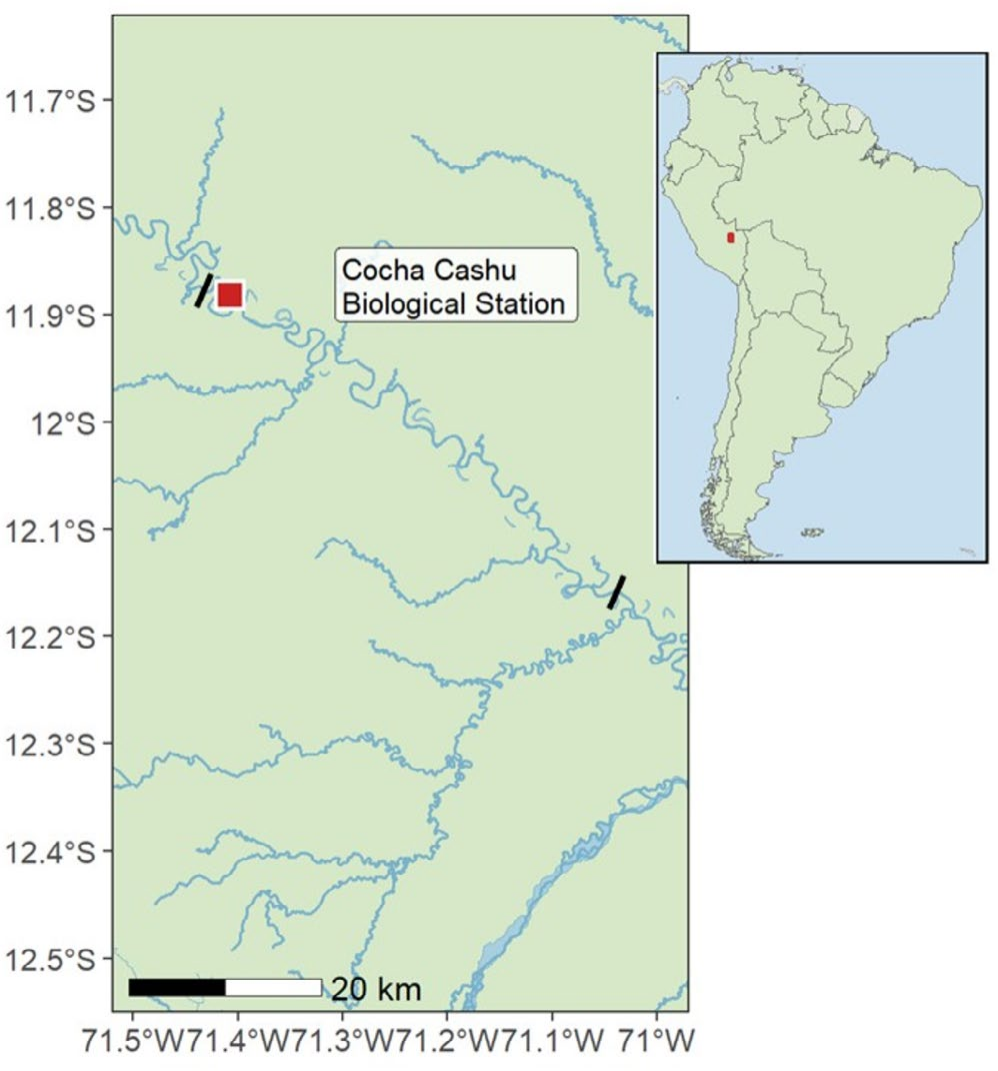
(Inset map right) Location of the study in South America. (Left) A map of the study area within Manu National Park in the Madre de Dios Region of southeastern Peru. The study area encompasses 47 km of the Rio Manu and is highlighted on the map with black dashes. The red square indicates the location of Cocha Cashu Biological Station (11°54′ S 71°22′ W) where the study was based.

Fieldwork was conducted along a 47‐km stretch of the Manu River within Manu National Park, in the Madre de Dios Region of southeastern Peru (11°51′ S, 71°19′ W; Figure 2) during the 2017 and 2018 breeding seasons. The Manu River is a white‐water tributary originating in the Andes Mountain Range and is characterized by high suspended sediment loads and elevated aquatic productivity (Fittkau et al. 1975; Osorio et al. 2011). During the dry season, declining water levels expose numerous sandy riverbanks and oxbow lake margins suitable for ground‐nesting waterbirds (Robinson and Terborgh 1997). Depending upon annual river water levels, a range of 108–118 riverbanks are available as nesting grounds within the study area. Fieldwork was conducted under authorization from the Peruvian Ministry of the Environment through the National Service of Natural Areas Protected by the State (Permit N13‐2017‐SERNANP‐PNM‐JEF) and under approval from the University of Oklahoma Institutional Animal Care and Use Committee (R18‐020).

### 2.2 Nest Monitoring and Reproductive Parameters

Nest searches were conducted by boat from 01 July–01 September 2017 to 17 July–30 September 2018. Nesting activity was identified by observing courtship behavior, scraping, incubation postures, and anti‐predator displays. For each nest located, we recorded date, time, and GPS coordinates. Nests were monitored until hatching, failure, or 28 days post‐hatch with unknown fate. The 28 day cutoff is based upon previous Black Skimmer studies (see Table 1 for references).

**TABLE 1:** Life history trait comparison for Manu and other Black Skimmer ( *Rynchops niger* ) populations across the Americas.

Parameter	Manu mean (SD), [95% CI]	North American weighted mean, 95% CI	North America data	Brazilian Amazon and Pantanal data	Literature sources^a^
Clutch size (egg per clutch)	3.11 (0.72) [2.97, 3.27]	3.03 (2.24), [−1.71, 7.76]	Range 1.3–3.70	2.29 ± 0.92 [18], 2.83 ± 0.59 [19]	[1, 2, 3, 4, 5, 18, 19]
Incubation bout (min)	7.30 (1.71) [6.71, 7.88]		25–57	20.6 (15.65)	[6, 7, 18]
Egg incubation period (days)	23.61 (3.02), [22.59, 24.63]		Range 20–24	21.6 (1.6)	[1, 3, 8–11, 18]
Daily nest survival rate (DSR)	0.967 (2017) 0.983 (2018)		0.7–0.982, variable with location		[1, 3, 9, 10, 11, 12, 19]
Hatch success	38% (2017) 46% (2018)		36%–88%, variable		[3, 5, 8–11, 13, 16, 17]
Provisioning rate (fish/young/h)	3.58 (1.35) [3.02, 4.14]		0.12–1.5		[1, 3, 8, 13, 16]
Fledge rate (days)	18 (2.3) [17.89, 18.11]		28–31.5 days	27	[1, 3, 7, 13, 18]
Growth rate constant (K)	0.19 (m) 0.31 (f)		0.22 (m), 0.19 (f)—VA, 0.18—VA, 0.28—CA	0.12	[3, 14–16, 18]
Upper asymptote	299 g (m) 239 g (f)		326 g (m), 260 g (f‐VA), 366 g (m‐CA), 271 g (f‐CA)		
Inflection point (I) (days)	14 (1.7, f) 23 (3.6, m)		CA‐11.73 (f), 4.25 (m), VA 15 (m & f)		[3, 14]
*t*_10–90_ (days)	23.1 (m), 14 (f)		20 (m‐VA), 23 (f‐VA), 15.7 (m & f‐CA)	37.7	[7, 14, 18]

^a^ Literature sources: [1] DePue (1974); [2] Safina and Burger (1983); [3] Erwin (1977a), [4] Gordon et al. (2000); [5] O'Connell and Beck (2003); [6] Gochfeld et al. (2020); [7] Schuchmann et al. (2022); [8] Quinn (1990); [9] Dinsmore et al. (2002); [10] Owen and Pierce (2013); [11] Brooks et al. (2014); [12] Custer and Mitchell (1987); [13] Gochfeld and Burger (1994); [14] Schew and Collins (1990); [15] Schew et al. (1994); [16] Gordon et al. (2000); [17] Groom (1992); [18] Schuchmann et al. (2018); [19] Krannitz (1989).

Annual hatching success was calculated as the proportion of monitored nests that produced at least one chick. A nest was considered successful if one or more eggs hatched. Causes of failure were categorized as flooding, predation, or other (e.g., abandonment, unknown fate). Mean clutch size, incubation period, and incubation bout duration (±SD) were calculated for each year. We used Mann–Whitney *U* analyses to compare seasonal differences in clutch size, incubation duration, and incubation bout duration.

Daily nest survival rate (DSR) was estimated using the Mayfield method (Mayfield 1961, 1975) to allow comparisons with older published studies. Exposure days were calculated for each nest from estimated laying date to fate. We assumed that eggs were laid at one day intervals unless directly observed, and one chick hatched per day. Hatch timing was inferred from clutch completion and chick presence when the exact hatch date was unknown. The incubation period was averaged to 22 ± 3 days based on our incubation study results.

DSR was calculated as follows:

$$\mathrm{DSR}=1-\frac{\mathrm{NF}}{E}$$

where *NF* = number of failed nests and *E* = total exposure days.

### 2.3 Adult Capture and Incubation Behavior

We selected 40 nests (*n* = 20 for 2017 and 2018) for detailed behavioral monitoring. One adult per focal nest was captured using a modified loop carpet trap (Goodenough 2022). Adults were banded with unique coded field‐readable plastic bands and measured for wing chord, mass, exposed culmen, and bill depth. Sex was assigned using established morphometric criteria, as males are consistently larger across measurements (Erwin 1977a; Schew and Collins 1990; Scherer et al. 2013).

Incubation behavior was recorded using E7000 action cameras (Akaso, China) paired with portable battery packs and 128‐GB microSD cards. Cameras were housed in waterproof camouflaged cases and positioned 2–5 m from the nest. Video recordings consisted of 15‐min clips spanning 6–8 h observation bouts. From these recordings, we quantified incubation bout duration as the interval between parental exchanges at the nest.

### 2.4 Chick Provisioning and Growth

Chicks were monitored from hatching through fledging or mortality. Video monitoring was conducted from Day 1 through Day 12 post‐hatch. As most of the river banks had only one skimmer pair, we assumed if a chick was observed present on a particular riverbank that it was an individual we previously assigned to the nest that was documented for each riverbank. We banded chicks with field‐readable bands to be able to follow their movements outside of the nest. Once chicks were mobile (typically Day 7–12), provisioning observations were conducted from a boat at a mean distance of 100 m using an 80‐mm spotting scope.

Provisioning rate was calculated as follows:

$$\text{Provisioning rate}=\frac{\text{Total prey items delivered}}{\text{Number of chicks}\times \text{Total observation hours}}$$

Provisioning observations totaled 10,058 min across both seasons. Rates were calculated per nest and averaged by year, then pooled to obtain an overall mean provisioning rate (±SD).

Non‐flighted chicks were measured twice weekly for: unflattened wing chord (mm), body mass (g), emergence and length of primary flight feathers, rectrix development, and total head (mm). It was not possible to obtain permission to collect genetic samples in Manu National Park. Therefore, sex was assigned to chicks based on morphometric measurements taken at 10–17 days of age, following Schew and Collins (1990). Male skimmer chicks at 17 days of age are on average about 100 g larger than females (Schew and Collins 1990). Additionally, chick bill length of both the upper mandible and lower mandible was monitored from day 10 onward, as the elongation of the lower mandible beyond the upper mandible—a dimorphic trait in Black Skimmers—was used as a secondary indicator of sex. Male chicks began showing this bill elongation from day 10, whereas females did not exhibit this morphological change until they fledged, providing an independent cross‐check of morphometric sex assignments. We considered chicks with a larger mass and an asymmetric bill length at age 10–17 days to be males. Fledging or attaining flight was defined as sustained flight exceeding 500 m. Growth trajectories were modeled using a logistic growth function (Ricklefs 1969):

$$W\left(t\right)=\frac{A}{1+e^{K\left(t-t_{1}\right)}}$$

where $W\left(t\right)$ = mass at age *t*, *A* = asymptotic mass, *K* = growth rate constant (days^−1^), and $t_{1}$ = age at inflection point.

We calculated: the growth rate constant (*K*), age at inflection point, and time from 10%–90% of asymptotic mass ($t_{10–90}=4.4/K$). Because sexual dimorphism emerges early in development (starting at 12 days, Schew and Collins 1990), growth curves were modeled separately for males and females.

### 2.5 Comparative Population Analysis

To evaluate whether reproductive traits observed in Manu were consistent with observations from other populations, we conducted a literature review using Web of Science. Search terms included “Black Skimmer,” “ *Rynchops niger* ,” “Rynchops niger cinerascens” and reproductive parameters that included “clutch size,” “daily nest survival,” “growth rate,” “incubation,” “provisioning,” and “fledge” or “fledging.”

We identified 26 relevant publications, of which eight populations met inclusion criteria (reported sample size, mean, and variance, and used comparable methods). Several populations had more than one citation referencing them. Six populations were located in North America and two in South America (Brazilian Amazon and Pantanal). Where possible, weighted means and 95% confidence intervals were calculated for clutch size, incubation period, daily nest survival, provisioning rate, growth rate constant, and fledging age. Because not all traits were reported for all populations, comparisons were parameter‐specific. This comparative framework allowed us to evaluate whether patterns observed in the Manu population—particularly incubation behavior, provisioning rate, growth constant, and fledging age—were consistent with environmentally structured variation in parental investment and offspring development across ecological contexts.

## 3 Results

### 3.1 Manu River Population

Unlike North American populations that can nest in colonies up to 250+ breeding pairs (Gochfeld et al. 2020), the Manu Skimmers nested singly per 0.5–1 km river bank with the exception of one location that consisted of 12 pairs down river near the convergence of the Boca Manu and Manu River where Manu National Park begins. Anecdotally, we believe this is also a staging area for skimmers that are transiting out of the park toward non‐breeding grounds along the Peruvian and Chilean coasts (Davenport et al. 2016).

Daily nest survival rates were similar with 0.968 (2017) and 0.971 (2018). Despite similar DSR values, the dominant sources of nest failure in Manu differed between years. In 2017, river flooding accounted for 60.9% of nest failure, whereas predation was the primary cause of nest failure in 2018 (55.3%), followed by flooding (41.2%). Combined, flooding and predation explained greater than 95% of nest failures in both seasons, indicating that reproductive failure was largely attributable to these two factors.

Mean clutch size for the Manu population was 3.17 ± 0.74 eggs per clutch (95% CI: 2.95–3.27) and did not vary across breeding season (Mann–Whitney *U* test, *U* = 1102.5, *p* = 0.758, effect size *r* = −0.034). Incubation period varied between years in Manu (Mann–Whitney *U* test = 84.0, *p* = 0.013, effect size *r* = 0.481), with 2018 nests having a longer incubation duration than 2017 nests. The 2017 incubation period was 22.44 ± 2.85 days (median = 22, range 19–29 days). The 2018 incubation period was 24.78 ± 2.78 days (median 25, range 18–29).

Incubation bout length did not differ significantly between years (Mann–Whitney *U* = 141, *p* = 0.571, *r* = −0.13 and Welch *t*‐test *p* = 0.488, *t*(13.6) = 0.71, Cohen's *d* = 0.30) and averaged 7.33 ± 1.84 switches/h (range = 4.9–12.0) across years. We were concerned that there may be a potential confounding effect. We assessed whether individuals that were monitored for different lengths of time could influence observed switching rates. A Spearman correlation between time monitored and switches per hour found a weak negative trend with longer monitoring windows associated with slightly lower rates, but it was not significant (Spearman *r* = −0.314, *p* = 0.066, Pearson *r* = −0.278, *p* = 0.105).

### 3.2 Chick Provisioning

Chick provisioning was monitored from late June through mid‐September in both years. Video observations totaled 9146 min across 25 nests (6 nests in 2017; 19 nests in 2018), spanning chick ages Day 1–12. An additional 912 min of focal observations were conducted on chicks aged 12–17 days, yielding 10,058 min of total provisioning observation. Across monitored nests, 641 prey items were delivered to chicks. Parents brought food to chicks regularly throughout the day, and these delivery rates remained consistent across the time period we monitored chick development. In 2017, six nests were monitored through Day 12, though only two nests retained chicks beyond Day 12. Mean provisioning rate for 2017 was 3.32 ± 1.41 fish per chick per hour. In 2018, 19 nests were monitored through at least Day 6 and 10 nests through Day 12; mean provisioning rate was 3.67 ± 1.36 fish per chick per hour. The two seasons were not significantly different (Mann–Whitney *U* = 46.5, *p* = 0.522, Cohen's *d* = −0.26). When pooled across seasons, the overall provisioning rate was 3.58 ± 1.35 fish per chick per hour. When adjusted for brood size, pairs with two chicks provisioned at nearly double the raw rate of single‐chick broods (6.80 vs. 3.64 prey/h), and this is highly significant (Mann–Whitney *U* = 5.0, *p* = 0.002, *r* = 0.90).

### 3.3 Chick Growth and Fledging

We collected morphometric data from 71 chicks (14 in 2017; 57 in 2018). Mean wing chord growth was 7.07 ± 3.67 mm/day, and mean mass gain was 8.43 ± 5.0 g/day. More chicks survived to fledge in 2018 (*n* = 7) than in 2017 (*n* = 3). Logistic growth modeling revealed sex‐specific differences in growth trajectories (Table 2). While only 10 chicks were documented to fledge, the growth model data include all chicks that were measured at least twice within the monitored period (*n* = 14 for 2017 and *n* = 21 for 2018). Female chicks (*n* = 13) exhibited a higher growth rate constant (*K* = 0.31) than males (*n* = 22, *K* = 0.19). Females reached the inflection point of growth at 14 ± 1.7 days, whereas males reached inflection at 23 ± 3.6 days. Chicks attained sustained flight at a mean age of 18 ± 2.3 days (95% CI: 16.0–20.3 days).

**TABLE 2:** Growth parameter comparison for Manu, California, and Virginia Black Skimmer ( *Rynchops niger* ) populations.

Parameter	Manu NP	California	Virginia
Growth rate constant (K)	0.19 (m), 0.31 (f)	0.28	0.22 (m), 0.19 (f)
Upper asymptote (I)	299 g (m), 239 g (f)	366 g (m), 271 g (f)	326 g (m), 260 g (f)
Inflection point (days)	14 (f), 23 (m)	11.73 (f), 14.25 (m)	15 (both sexes)
*t*_10–90_ (days)	23.1 (m), 14 (f)	15.7 (both sexes)	20 (m), 23 (f)

### 3.4 Comparative Population Patterns

Daily nest survival rates reported for other populations in North and South America ranged from 0.70 to 0.98 (Table 1). The Manu DSR values (0.97–0.98) fell within this range and were comparable with 95% confidence intervals reported for other populations. The highest non‐Manu DSR (0.98) was reported from a tropical population in Pará, Brazil.

Across all surveyed populations in North America, nest predation was the most frequently reported cause of failure, followed by coastal flooding. Coastal colonies experienced losses associated with spring tides and storm surges, whereas inland flooding was rarely reported outside of the Manu system. The Manu population experienced substantial nest loss due to flood events (41%–61% of failures). Unfortunately, the only other South American colony comparison that we have is a population in Brazil where flash flooding is uncommon, so comparisons to other locations in South America vulnerable to flash flooding are limited (Schuchmann et al. 2022).

Mean clutch size for the Manu population (3.11 ± 0.72) was comparable to values reported from Texas and Virginia and Brazilian populations. Incubation period (22.25 ± 2.4 days) was likewise similar to values reported across North and South America. Incubation bout duration in Manu (7.3 ± 1.7 min) was markedly shorter than those documented in a North Carolina population (24–57 min) and shorter than limited data from Brazil (Schuchmann et al. 2022, 20.6 ± 15.65 min), although sample sizes for comparison populations were small.

The Manu population stood out among the other colony studies in that Black Skimmers breeding on the Manu River had shorter incubation bouts (also known as nest switches) and delivered food more frequently than coastal populations. Incubation bout duration in Manu was 7.3 ± 1.7 min—much shorter than durations documented in a North Carolina population (24–57 min) and in a limited data set from Brazil (20.6 ± 15.65 min). As for provisioning rates, non‐Manu populations delivered 0.12 to 1.5 prey items per chick per hour, whereas Manu provisioning averaged 3.58 ± 1.35 items per chick per hour. Growth rate constants from the Manu population were comparable to or exceeded those reported from California and Virginia. However, age at fledging differed substantially when comparing Manu with other skimmer populations (Table 1). Manu chicks achieved sustained flight at 18–20 days post‐hatch, whereas North American populations typically report fledging between 28 and 31 days. Fledging success was low across all populations examined (11%–33%). South American populations, including Manu, tended toward the lower end of this range (3%–14%).

## 4 Discussion

We found that clutch size, incubation period, daily nest survival, and hatch success in the Manu River population were broadly similar to those reported for other populations across the Americas. In this study, incubation bout duration, chick provisioning rates, growth trajectories, and fledge rate differed markedly. These results suggest that the most pronounced variation occurs in traits directly influencing the duration of the vulnerable developmental stage rather than in initial reproductive investment per se. Skimmers in Manu exhibited shorter incubation bouts, had substantially higher provisioning rates, had accelerated chick growth, and fledged at 18–20 days—approximately 10 days earlier than typically reported for North American populations (Table 1 and references within). Taken together, these coordinated shifts in parental behavior and offspring development suggest that balancing parental energetic expenditure against offspring growth rate, and minimizing the duration of stage‐specific vulnerability against the costs of accelerated development are being resolved differently in Manu than in North American populations. These observed patterns of elevated provisioning, accelerated growth, and early fledging could reflect phenotypic plasticity, local adaptation, or some combination of both.

The accelerated chick growth and earlier fledging observed in Manu are most directly explained by the exceptional prey availability along this productive white‐water river system, which enabled parents to sustain provisioning rates well above those documented in other populations. In temperate systems, provisioning rates of approximately 0.5 fish per chick per hour are often considered typical, with rates approaching 1.5 items per chick per hour documented during seasons of high prey availability (DePue 1974; Erwin 1977a; Quinn 1990; Gochfeld and Burger 1994; Gordon et al. 2000). In contrast, Manu adults delivered an average of 3.6 prey items per chick per hour. These values suggest that food resources along the Manu River were not limiting during the chick‐rearing period and that parents had the capacity to allocate energy toward rapid offspring growth. Faster growth is broadly advantageous for offspring survival regardless of the specific mortality regime, and the high food availability in Manu appears to have permitted parents to achieve what would likely be beneficial in any system. Whether recurrent flash flooding and predation pressure further reinforce selection for rapid development, or whether early fledging is primarily a consequence of superior nutritional conditions, cannot be determined from this study alone.

The elevated provisioning rates documented in Manu could reflect differences in either prey quantity or prey quality relative to North American populations. White‐water rivers draining Andean headwaters support high aquatic productivity and fish biomass (Fittkau et al. 1975; Osorio et al. 2011), suggesting that prey abundance rather than prey energy content is the more likely driver of accelerated chick growth in this system. Skimmers are generalist piscivores that take small surface fish across a range of species, and prey composition is unlikely to differ so dramatically between freshwater and coastal systems as to account for the magnitude of the difference in provisioning rates observed here. Furthermore, if prey quality were the primary explanation, we would expect chick growth rates to be elevated without a corresponding increase in delivery frequency, yet both were observed simultaneously in Manu suggesting a prey abundance effect. Prey energy content was not measured in this study, and future work quantifying the caloric value of prey delivered across populations would help resolve this question.

Before comparing provisioning rates across populations, two methodological points are worth noting. First, provisioning observations in this study were conducted during daylight hours and therefore represent minimum estimates of total food delivery. Black Skimmers are known to forage nocturnally (Rojas et al. 1997; Gochfeld et al. 2020), and anecdotal evidence from North American colonies suggests that a meaningful proportion of chick feeding may occur at night, although this behavior remains poorly documented in the primary literature. To the extent that nocturnal provisioning occurs in the Manu population, the rates reported here would underestimate true delivery rates. However, because previous provisioning studies used for comparison also relied on daytime observation only, the relative differences reported here are directly comparable across populations. Further, the elevated chick growth rates documented in this study are consistent with high total food intake, providing independent support for the interpretation that provisioning in Manu was genuinely elevated regardless of any nocturnal contribution.

Second, provisioning rates at Manu were estimated using a combination of video recording and direct in‐person observation, whereas the North American studies used for comparison relied exclusively on in‐person observation. If video recording captures feeding events that an observer might miss, that is, brief or rapid prey deliveries, then Manu provisioning rates may be slightly inflated relative to North American estimates. However, this methodological difference would bias our results in a conservative direction: any overestimation of Manu provisioning rates would mean that the true difference between Manu and North American populations is smaller than reported, not larger as is observed in this study. Given that Manu provisioning rates exceeded North American values by more than three‐fold, it is unlikely that observer method alone accounts for the magnitude of this difference. We therefore consider the comparison across populations to be valid in its overall interpretation, while acknowledging that standardizing observation methods across future studies would strengthen cross‐population comparisons.

Flooding was a dominant source of nest failure in Manu, accounting for 41%–61% of losses across years. Although tropical storms and hurricanes in the southeastern United States represent an episodic and difficult‐to‐predict source of flooding risk for skimmers nesting along the Atlantic and Gulf coasts of North America—with peak hurricane season overlapping with the latter portion of the skimmer breeding season—coastal nesting sites are also subject to semi‐lunar tidal cycles that create recurring, predictable patterns of water level change that birds can anticipate behaviorally through nest site selection and timing of breeding attempts (Plaschke et al. 2019; Van De Pol et al. 2010; Maslo et al. 2016). By contrast, flash flooding along the Manu River is driven by episodic cold air incursions and upstream storm events in the Andes (Marengo et al. 1997; Garreaud 1999) that operate on no predictable cycle and can inundate active nests with little warning. The distinction we draw here is not that coastal flooding is entirely predictable, but that Manu skimmers face a flood regime that offers no recurring temporal signal that could be tracked or anticipated through behavioral adjustment. Such events create intense, stage‐specific mortality risk concentrated during the egg and early chick period. Fitness is therefore influenced not only by the probability of nest survival on any given day, but also by the total duration of exposure to catastrophic events. Accelerated development that reduces the length of the pre‐flight stage may then confer a meaningful survival advantage even when instantaneous daily survival rates are held constant.

Nest predation was also a substantial source of failure, particularly in 2018, and remains the most frequently reported cause of nest loss across the Black Skimmer's breeding range. Whether predation pressure produces the same directional shift in developmental timing as flood‐driven mortality is difficult to determine from this study, as both sources of mortality co‐occur at Manu and cannot be experimentally separated. However, there are theoretical reasons to expect their effects on parental behavior to differ. Predation risk is most effectively reduced through behavioral responses such as altered nest attendance, nest site selection, and anti‐predator vigilance, whereas the catastrophic and indiscriminate nature of flash flooding may more directly favor accelerated chick development as the primary means of reducing stage‐specific vulnerability. In Manu, where both predation and flooding occur, the recurring and catastrophic nature of flood events, accounting for 41%–61% of nest failures across years, may represent the stronger selective pressure on developmental timing, though this interpretation remains speculative without data from populations experiencing flooding and predation independently.

Chick mortality was concentrated in the early post‐hatching period, with a two‐fold reduction in chick numbers observed within the first week after hatching. This pattern is consistent with the general principle that mortality rates are highest early in life in birds (Caughley 1966; Stearns 1992), and with evidence from related larid species that predation risk decreases as chicks become older and more mobile (Chokri et al. 2023). Erwin (1977a) similarly documented that fledging success in Black Skimmers was very low, with most mortality occurring before chicks attained flight capability. Although hatching success was relatively high at Manu, survival from hatching to fledging was low, suggesting that the chick stage rather than the egg stage represents the primary mortality bottleneck in this system. This pattern is consistent with flooding and predation acting most severely on young chicks that are flightless and confined to exposed riverbanks, limiting their ability to escape rising water or approaching predators. Under these conditions, any reduction in the duration of the pre‐flight period would be expected to reduce cumulative exposure to these stage‐specific mortality risks.

Although flooding, predation, and prey availability are emphasized here as primary drivers of accelerated chick development in Manu, other ecological differences between this population and North American colonies may also contribute to observed variation. In particular, colonial nesting in North American populations can involve density‐dependent dynamics including resource competition among chicks, chronic social stress, and risk of conspecific interference that are largely absent in the Manu system. Such stressors are known to affect growth rates in colonial waterbirds, though the direction of this effect is not uniformly negative. Savoca et al. (2011) found that Herring Gull chicks reared in more dense sub‐colonies actually grew faster than those in less dense colonies, suggesting that density effects depend heavily on ecological setting. In Manu, skimmer pairs nest in relative isolation. However, this study was not designed to isolate the relative contributions of density‐dependent effects versus flood‐driven selection on developmental timing. To tell these two explanations apart, future studies would need to compare chick growth and stress responses across populations where colony density and flood risk vary independently of one another.

Growth modeling further supports our interpretation of plasticity, although sample sizes limit the strength of cross‐population comparisons. When sexes were pooled, Manu chicks exhibited elevated growth constants and attained flight at a mean of 18 ± 2.3 days, substantially earlier than the 28–31 days reported for North American populations (Depue 1974, Erwin 1977a; Gochfeld and Burger 1994; Schew and Collins 1990; Schew et al. 1994). Sex‐specific modeling suggested that female chicks had higher growth constants and reached inflection points earlier than males (*K* = 0.31 vs. *K* = 0.19; inflection points 14 ± 1.7 days vs. 23 ± 3.6 days), a pattern consistent with data from a California population (Schew and Collins 1990), suggesting this may reflect a broader pattern of sex‐specific development in Black Skimmers rather than an artifact of the Manu sample.

The small number of chicks surviving to fledge (*n* = 3 in 2017, *n* = 7 in 2018) means these sex‐specific results should be interpreted cautiously and warrant confirmation with larger sample sizes. Chick sex was assigned using morphometrics at 15–17 days in conjunction with monitoring of bill morphology from day 12 onward, which provided an independent cross‐check on sex assignment. Retrospective verification against adult morphology was not possible given the delayed sexual maturity of this species and the limited duration of this study. We acknowledge this as a limitation and encourage future studies to incorporate genetic sexing of chicks where possible. Regardless of how sexes are partitioned, the earlier fledge rate in Manu chicks is a robust finding. The combination of high provisioning rates, elevated growth constants, and early flight capability suggests that parental behavior and chick developmental trajectories are aligned in a manner that shortens the window of vulnerability to stage‐specific mortality.

Adult morphometric data collected from breeding birds at Manu provide further context for interpreting the early fledging observed in this population. Mean mass and wingchord of Manu adults were comparable to or exceeded values from North American populations (Manu females: 247 ± 27 g, 381 ± 16 mm; Manu males: 354 ± 23 g, 426 ± 12 mm; North American females pooled: 262 ± 29 g, 365 ± 9 mm; North American males pooled: 325 ± 36 g, 398 ± 11 mm). Manu birds therefore do not appear to be a smaller subspecies, and the earlier fledging documented here does not appear to result in permanently smaller adults. This suggests that chicks continue growing after attaining flight, and that fledging earlier represents an acceleration of the transition out of the most vulnerable stage of development rather than a permanent reduction in final body size. Tracking chicks after fledging and measuring any costs of elevated provisioning effort to parental condition or future survival were beyond the scope of this study and remain priorities for future research.

Definitively distinguishing phenotypic plasticity from local adaptation would require a reciprocal transplant study or a common garden experiment tracking offspring of Manu and North American birds under ideal conditions that is logistically impractical for a species undertaking long‐distance migrations. In the absence of such data, the patterns observed here are interpreted as consistent with, though not proof of, environmentally mediated phenotypic plasticity. Hemispheric genetic differentiation has been documented in the Black Skimmer (Mariano‐Jelicich and Madrid 2014; Goodenough et al. 2024), suggesting a potential for regional adaptation. If divergence in flood regimes and food availability has been persistent across generations in Manu, directional selection on skimmer provisioning capacity and chick developmental rate is plausible. The absence of variation in clutch size and incubation duration, coupled with pronounced differences in provisioning and growth, supports the interpretation that at least part of the response may be plastic. Plastic responses to food availability and flood risk would be expected to act on downstream behavioral and developmental traits, not on upstream investment decisions like clutch size, which tend to be less responsive to local environmental conditions and more consistent across populations (Stearns 1992; Ricklefs and Wikelski 2002). More research would be needed to disentangle genetic differentiation from environmentally mediated reaction norms (Westneat et al. 2011).

It is also worth considering what the similarity in clutch size between Manu and North American populations tells us, given what we might expect based on latitude alone. Tropical bird populations generally tend to have smaller clutch sizes than their temperate counterparts, so finding no difference between Manu and North American skimmers may be informative. It could suggest that Manu birds are reproducing at a faster rate than would be typical for a tropical population, possibly because abundant food resources and strong pressure to fledge chicks quickly push them toward greater reproductive effort. Under this interpretation, the traits that did not differ between populations are just as ecologically informative as those that did.

The ecological context of the Manu River, high aquatic productivity combined with recurrent flash flooding during the breeding season, appears to create conditions under which accelerated chick development is both feasible and advantageous. Where food availability permits higher provisioning rates, parents may resolve the tradeoff between self‐maintenance and reproductive investment in favor of rapid offspring growth, thereby reducing exposure to catastrophic mortality events. In environments where food is limited, such elevated effort may not be achievable, extending chick vulnerability across the rearing stage and potentially increasing cumulative mortality risk (Lok et al. 2014). The Manu population represents a single inland tropical system, and we urge caution when extrapolating these results to other riverine populations, as the influences on reproduction may vary considerably across systems. Additional data from other flash‐flood‐prone skimmer breeding colonies would be required to determine whether accelerated development is a general feature of such systems.

Life‐history studies spanning broad geographic gradients within a single species remain uncommon, particularly for tropical populations. By combining reproductive behavior, provisioning dynamics, and growth modeling within a comparative framework, this study demonstrates that substantial variation in developmental timing can occur within a species without changes in clutch size or incubation duration. Such a pattern is consistent with flexible adjustments of parental care allocation and growth trajectories in response to localized mortality regimes rather than wholesale divergence in life‐history strategy.

Future changes in hydrology associated with climate change and land‐use alteration are likely to alter flooding regimes in both coastal and inland systems. Global predictions for climate change suggest increased sea level rise, marked changes in precipitation patterns, and increased frequency of storm events, all of which will continue to influence bird population demographics (Plaschke et al. 2019; Bayard and Elphick 2011). Flooding regimes are already being altered globally due to climate change (Ledger et al. 2012; Royan et al. 2015) and continued land‐use change—including coastal development, hydroelectric dam construction, and deforestation on inland mountain slopes—can synergistically create disastrous environmental conditions for wildlife. Dams are known to impact the ecology of pulse‐flood systems by altering hydrology and associated ecological processes (Finer and Jenkins 2012), and deforestation on mountain slopes can destabilize soils and increase flash flood probability (Stoffel et al. 2016). At a minimum, altered flow regimes can create phenological mismatches with prey that in turn influence the tradeoffs birds use to mitigate reproductive impacts (Royan et al. 2015; Zeigler and Fagan 2014). Additional research on avian responses to flood dynamics is crucial to understanding the capacity of nesting birds to adapt or acclimate to ongoing environmental change, and to inform management practices in the face of continued hydrological alteration.

## Author Contributions

**Katharine S. Goodenough:** conceptualization (lead), data curation (lead), formal analysis (lead), funding acquisition (supporting), investigation (lead), methodology (lead), project administration (lead), writing – original draft (lead), writing – review and editing (lead). **Torbjørn Haugaasen:** conceptualization (supporting), formal analysis (supporting), investigation (supporting), methodology (supporting), writing – original draft (equal), writing – review and editing (equal). **Eli S. Bridge:** conceptualization (supporting), formal analysis (supporting), funding acquisition (lead), investigation (supporting), methodology (equal), writing – original draft (equal), writing – review and editing (equal).

## Funding

This work was supported by a National Science Foundation Rules of Life grant (1840230), The Oklahoma Biological Survey, and a Waterbird Society Research Grant 2018.

## Conflicts of Interest

All co‐authors have seen and agree with the contents of this manuscript. This submission is original work and is not under review at any other journal. We have no conflict of interest to disclose.

## Data Availability

Data collected for this research is archived at Open Science Framework and available for review by editorial board and manuscript reviewers. The repository includes both data and R code used to analyze data (https://osf.io/en9kx/?view_only=ac623ac217cb4b608294ccdb22ad3f87).
